# Identification of S1PR3 gene signature involved in survival of sepsis patients

**DOI:** 10.1186/s12920-021-00886-2

**Published:** 2021-02-06

**Authors:** Anlin Feng, Wenli Ma, Reem Faraj, Gabriel T. Kelly, Stephen M. Black, Michael B. Fallon, Ting Wang

**Affiliations:** 1 Department of Internal Medicine, College of Medicine-Phoenix, University of Arizona, 475 N. 5th Street, Phoenix, AZ 85004 USA; 2 Department of Medicine, College of Medicine-Tucson, University of Arizona, Tucson, AZ USA

**Keywords:** Microarray, S1PR3, Sepsis, S3MS

## Abstract

**Background:**

Sepsis is a life-threatening complication of infection that rapidly triggers tissue damage in multiple organ systems and leads to multi-organ deterioration. Up to date, prognostic biomarkers still have limitations in predicting the survival of patients with sepsis. We need to discover more prognostic biomarkers to improve the sensitivity and specificity of the prognosis of sepsis patients. Sphingosine-1-phosphate (S1P) receptor 3 (S1PR3), as one of the S1P receptors, is a prospective prognostic biomarker regulating sepsis-relevant events, including compromised vascular integrity, antigen presentation, and cytokine secretion. Until now, no S1PR3-related prognostic gene signatures for sepsis patients have been found.

**Methods:**

This study intends to obtain an S1PR3-associated gene signature from whole blood samples to be utilized as a probable prognostic tool for patients with sepsis.

**Results:**

We obtained an 18-gene S1PR3-related molecular signature (S3MS) from the intersection of S1PR3-associated genes and survival-associated genes. Numerous important immunity pathways that regulate the progression of sepsis are enriched among our 18 genes. Significantly, S3MS functions greatly in both the discovery and validation cohort. Furthermore, we demonstrated that S3MS obtains significantly better classification performance than random 18-gene signatures.

**Conclusions:**

Our results confirm the key role of S1PR3-associated genes in the development of sepsis, which will be a potential prognostic biomarker for patients with sepsis. Our results also focus on the classification performance of our S3MS as biomarkers for sepsis, which could also provide an early warning system for patients with sepsis.

## Background

Sepsis is a severe and life-threatening clinical syndrome with a primary systemic bacterial infection. Alarmingly, the occurrence of sepsis is rising with a 17% increase between 2000 and 2010 [1]. At the same time, the mortality of sepsis has risen unacceptably high with a 31% increase between 1999 and 2014 [2]. This elevation in mortality rate is partially due to the complexity of its immunological syndrome and multiorgan involvement [3]. An effective prognostic biomarker that can predict the clinical outcome of sepsis patients appropriately is in high demand in clinical practice.

Biomarkers for sepsis may be utilized as a diagnostic or prognostic tool. Circulating proteins including pro-inflammatory cytokines, complement proteins, or immunosuppressive phase proteins, have previously been identified as single biomarkers for sepsis [4, 5]. However, single biomarkers lack specificity and sensitivity as prognostic tools for sepsis patients. The biomarkers containing multiple genes deliver better prognostic power. Several studies [6, 7] attempted to combine pro-inflammatory and anti-inflammatory markers and is the method to most likely succeed in predicting the disease development of sepsis patients. Meanwhile, a few gene signatures associated with immune responses in sepsis were also found in recent years. Emma E Davenport et al. [8] unified genomics approach and heterogeneity in sepsis to group patients by different immune responses. Miguel Reyes et al. [9] identified a unique CD14^+^ monocyte state which is inflated in sepsis patients. In general, biomarkers to characterize immune status are powerful tools for predicting the development and progression of sepsis.

Sphingosine 1-phosphate (S1P) is a bioactive lipid with specific targets and functions in both intracellular and extracellular environments. Following release from the cell, S1P acts as a ligand upon binding to five subtypes of S1P receptors (S1PRs) 1–5 which belong to the G protein-coupled receptor (GPCRs) family, triggering many receptor-dependent cellular signaling pathways. Among all these five S1PRs, S1PR3 regulates many parts of the vascular barrier and inflammatory responses in several pathological disorders related to the sepsis mediated pro-inflammatory response [10, 11]. S1PR3 is mainly expressed in the cardiovascular system, lungs, kidney, and spleen organs [12]. The S1P ligation of S1PR3 could affect various organ system functions such as vascular permeability signaling [13], heart rate, and blood pressure [14].

S1P-S1PR3 axis played a pivotal role in sepsis. Two independent studies have reported that S1P levels in the blood are reduced in sepsis, and its expression correlates with the severity of sepsis [15, 16]. S1PR3′s function in sepsis has been studied by multiple groups. Niessen [17] et al. suggested that the S1P–S1PR3 axis regulates late-stage inflammation amplification in sepsis. Hou et al. [18] also showed that S1PR3 signaling is bactericidal because it is related to the function of monocytes and the deficiency of S1PR3 could therefore increase susceptibility to sepsis, S1PR3 expression levels were upregulated in monocytes from sepsis patients. Higher levels of monocytic S1PR3 drove the innate immune response against bacterial infection and highly associated with preferable outcomes. S1PR3 is also noted to be significantly upregulated in acute respiratory distress syndrome [19], a pulmonary consequence of sepsis, suggesting a key role in inflammation regulation. Therefore, S1PR3 is essential for survival in sepsis, and S1PR3-regulated cell signaling pathways in sepsis may offer a novel role in therapy. The S1P-S1PR3 signaling may have the predictive power for estimating the activity of host innate immune responses and subsequent pathogen elimination.

We hypothesize that multiple S1PR3-related genes, in combination with pro- and anti-inflammatory cytokines, could correlate with clinical outcomes in sepsis patients. We desire to find those genes regulated by S1PR3 which may reflect the clinical outcome of sepsis patients. To achieve this aim, we examined whole blood gene expression in two standalone cohorts from Gene Expression Omnibus (GEO) and identified a gene signature of 18 genes significantly associated both with S1PR3 and sepsis survival. Our results propose that S1PR3-associated genes may expand the outcome prediction in sepsis.

## Methods

### Sepsis datasets

Two sepsis datasets with whole blood samples (GSE54514 and GSE33118) from the GEO database (https://www.ncbi.nlm.nih.gov/gds/) were chosen as our research subjects (Table 1). The discovery cohort GSE54514 contains whole blood transcriptome data of 35 survivors and non-survivors of sepsis, samples were collected daily for up to 5 days from sepsis patients. For validation cohort GSE33118, whole blood samples from 20 sepsis patients were tested before specific treatment.

**Table 1:** S1PR3-related gene signatures

Genes	logFC	FDR	Weight
MAPK1	− 0.621296141	0.003266485	− 1
SPHK2	− 0.406384121	0.002158621	− 1
S1PR4	− 0.54570687	0.021735813	− 1
MAPK3	− 0.575454873	0.002138805	− 1
AKT1	− 0.694671537	0.002092615	− 1
APP	− 0.357732416	0.01728323	− 1
GNAQ	− 0.362241659	0.017315486	− 1
CXCL16	− 0.833227304	0.027856529	− 1
C3AR1	− 0.71102393	0.019207385	− 1
FPR1	− 0.925845494	0.00265663	− 1
CXCL10	0.384326937	0.002895169	1
GNAI2	− 0.921587336	0.0000658	− 1
CXCR2	− 1.05300569	0.000703735	− 1
FPR2	− 0.836831366	0.021562632	− 1
RAC1	0.576528619	0.000000134	1
GABBR1	− 0.37486575	0.00477784	− 1
LPAR2	− 0.624948831	0.011513308	− 1
RHOA	− 0.43515886	0.02058016	− 1

*FC* fold change, *FDR* false discovery rate

Series matrix files containing pre-processed gene expression values were obtained from series GSE54514 (https://www.ncbi.nlm.nih.gov/geo/query/acc.cgi?acc=GSE54514) and GSE33118 (https://www.ncbi.nlm.nih.gov/geo/query/acc.cgi?acc=GSE33118). All chip probe sets in matrix files had been transformed into corresponding gene symbols by utilizing chip platform files.

### Identifying S1PR3-related sepsis gene signature

We detected the DEGs between 26 survivors and 9 non-survivors in the discovery cohort and set them as sepsis survival-related genes. The limma package [33] in R (version: 3.5.2) was used to identify DEGs in this study. The S1PR3-associated genes based on signaling pathways and associated proteins were confirmed by searching the STRING database (https://string-db.org/) [22]. We intersected the sepsis survival-related genes and S1PR3-associated genes, and these intersected genes were our S1PR3-related sepsis gene signature.

### KEGG pathway analysis and PPI network

We used different methods to display our gene sets’ functional profiles and interactions. The KEGG (Kyoto Encyclopedia of Genes and Genomes) pathway analyses were performed by the clusterProfiler [21] which was a visualization tool for analyzing functional profiles such as enriched pathways for gene clusters. We constructed the PPI network based on the STRING protein and protein interactions data being visualized by Cytoscape 3.5. Correlation matrix made by corrplot was used to highlight the most correlated genes in our gene table.

### Expression score and risk score

Each patient was allocated with an expression and risk score from gene expression and corresponding weight values of 18 genes. The linear formula corresponding to expression and risk score are:

$$\begin{aligned} expression\,score & = \mathop \sum \limits_{i = 1}^{n} \left( {{ }\frac{{e_{i} - \mu_{i} }}{{s_{i} }}} \right) \\ risk\,score & = \mathop \sum \limits_{i = 1}^{n} W_{i} \left( {{ }\frac{{e_{i} - \mu_{i} }}{{s_{i} }}} \right) \\ \end{aligned}$$

Here, n is the count of genes included in the gene signature in each dataset, W_i_ represents the weight of the ith gene (see in Table 1), e_i_ represents the expression level of the ith gene, and μ_i_ and s_i_ are the corresponding mean and standard deviation value for the ith gene among whole samples.

### Statistical analyses

R (version 3.5.0) was utilized to perform all the statistical calculations in this study. Receiver operating characteristic (ROC) curves and principal component analysis (PCA) was applied to prove the differentiating power of our S3MS on sepsis survival status. R package pROC (version 1.16.1) was used to visualize the ROC curve and compute the area under the curve (AUC). For PCA analysis, R built-in prcomp function was utilized to compute principal components, and the R package factorextra (version 1.0.7) to build the PCA plot. We set FDR < 0.05 as the statistically significant cutoff in this study.

## Results

### Identification of an S1PR3-related molecular signature (S3MS) associated with sepsis survival

Firstly, we identified all S1PR3-interactive proteins. STRING (https://string-db.org/) is an online biological database with acknowledged or predicted protein–protein interactions data. Utilizing the STRING database, we found a total of 226 S1PR3-related genes (Additonal file 1: Table S1) with a high confidence interaction score (interaction score > 0.7) in addition to all the active interaction sources, and all co-expression correlation r values of S1PR3 with S1PR3-related genes in discovery cohort had been shown in Additonal file 2: Table S2. Next, we defined all sepsis survival-related genes by setting the differentially expressed genes (DEGs) between the 26 survivors and 9 non-survivors as survival-related genes in the discovery cohort (NIH GEO GSE54514). 1078 up-regulated and 1134 down-regulated genes (false discovery rate [FDR] < 5% and fold change [FC] > 1.5) in non-survivors were found and characterized as sepsis survival-related genes (Additonal file 3: Table S3).

**Fig. 1:**
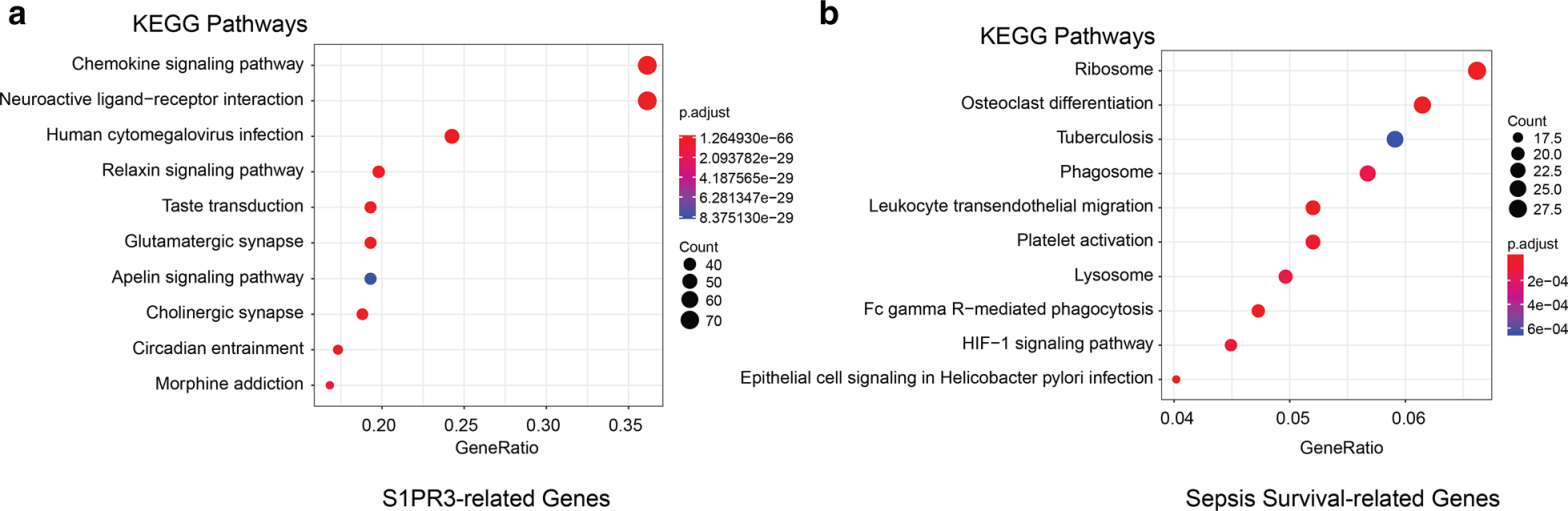
Enriched KEGG pathways. Enriched KEGG pathways among the S1PR3-related genes (**a**) and sepsis survival-related genes (**b**)

S1PR3-related genes from the STRING database and sepsis survival genes from our discovery cohort were then characterized. KEGG is a collection of databases for protein-coding gene functions, combining transcriptome with signaling pathways [20]. In this study, we applied an R package-clusterProfiler to detect enriched KEGG pathways amongst our genes [21]. The most significantly enriched KEGG pathways among S1PR3-interactive genes include chemokine signaling, neuroactive ligand−receptor interaction, and human cytomegalovirus infection (Fig. 1a) while the sepsis survival-related genes exhibited significant enrichment of ribosome, tuberculosis, and several immune-related pathways (Fig. 1b).

**Fig. 2:**
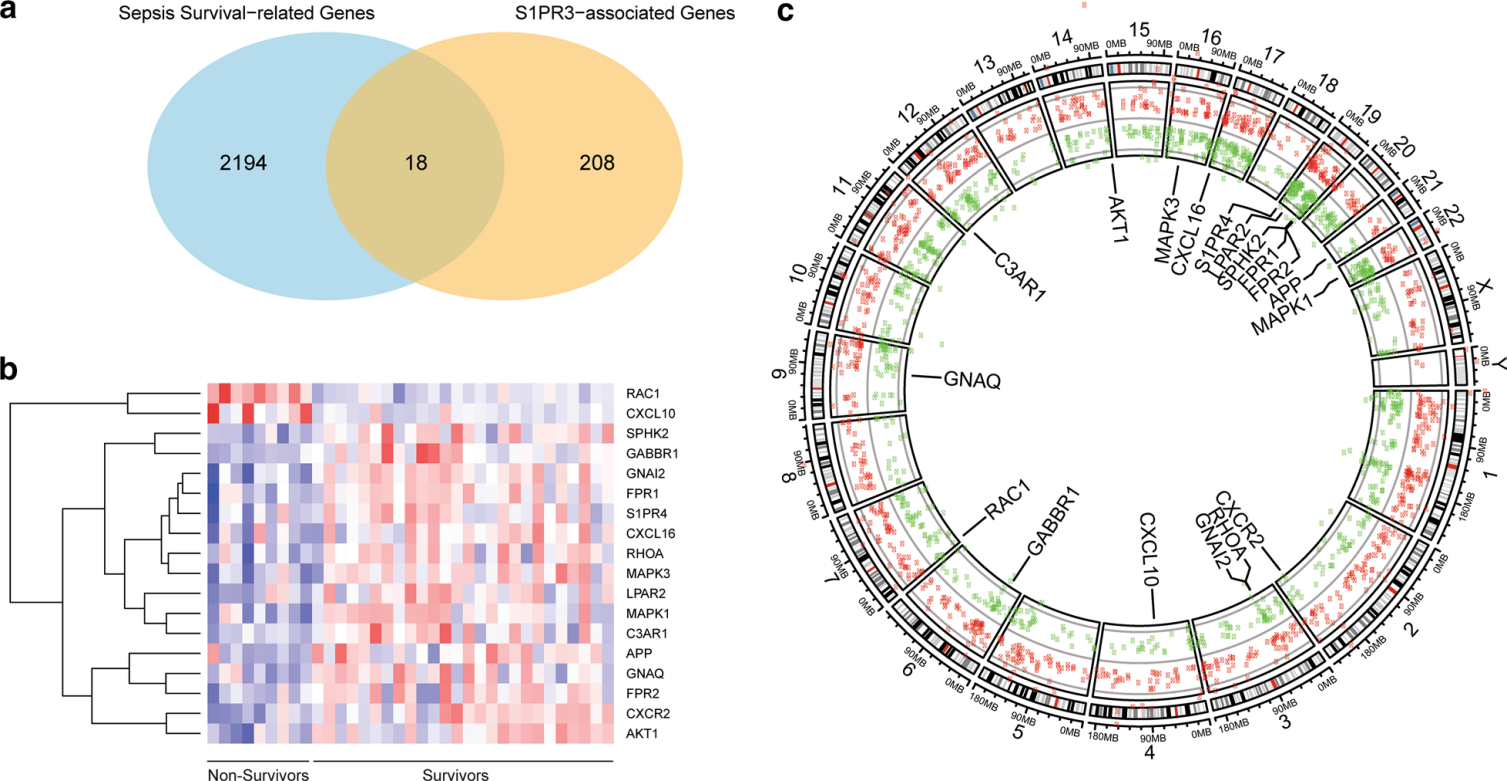
S3MS in sepsis patients. Venn plot (**a**) shows sepsis survival and S1PR3-associated overlapping genes. Heatmap (**b**) shows the S3MS gene expression in the discovery cohort. Red represents increased gene expression, while blue means decreased gene expression; (**c**) Genome circular plot for sepsis survival DEGs to illustrate all DEGs’ genome positions within chromosome (x-axis) and corresponding FC values (y-axis)

To find the connection between S1PR3 pathways and genes that affect sepsis survival, we intersected the S1PR3-related genes and sepsis survival-related genes and identified 18 overlapping genes (Fig. 2a). These 18 genes are classified as our sepsis gene signature derived from S1PR3-related genes in this study and defined as S1PR3-related molecular signatures (S3MS) (Table 1). Interestingly, within the human genome, this overlap is statistically significant (hypergeometric *p*-value < 0.05), suggesting S1PR3 related genes are significantly enriched among survival-associated genes in sepsis. The heatmap demonstrates that the 18 genes (S3MS) can discriminate non-survivors from survivors through different gene expression patterns (Fig. 2b). Graphical maps of genome sequence provide a method to rapidly look at the features of specific genes. A chromosome-based circular genome plot was utilized to illustrate all DEGs’ genome positions within chromosome (x-axis) and corresponding FC values (y-axis). Using a circular genome plot (Fig. 2c), we found that the 18 genes scattered in different genome regions suggesting that these genes are enriched in key pathways but not genetically linked due to chromosomal locations.

**Fig. 3:**
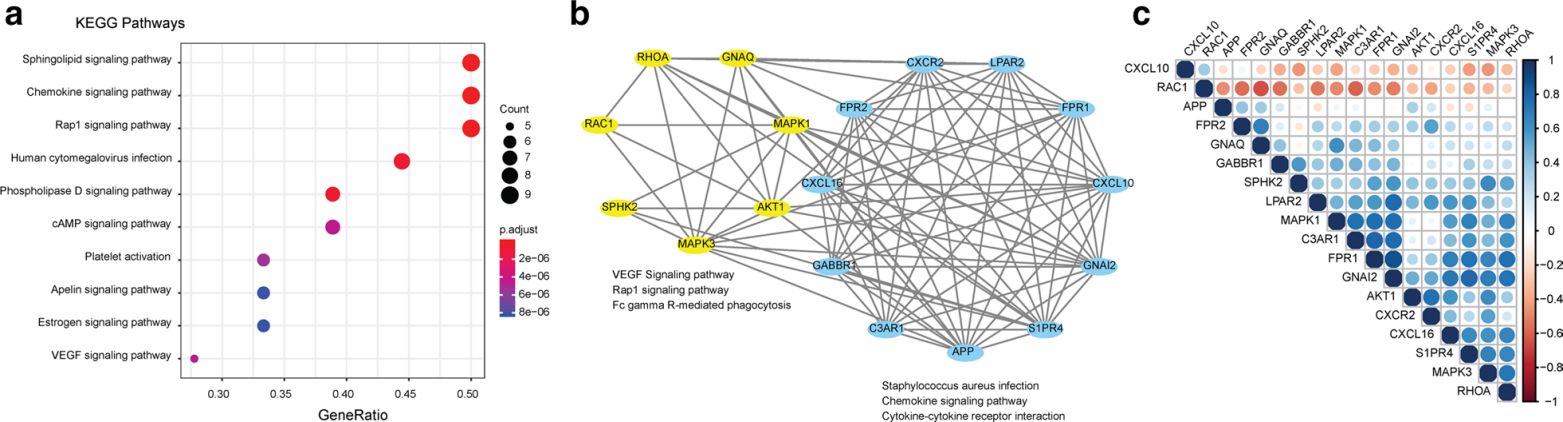
Biological characteristics of S3MS. Enriched KEGG pathways (**a**), PPI (**b**) and pairwise Pearson correlations matrix (**c**) among the S3MS

Next, we characterized the biological function and interaction of these 18 genes in the S3MS. KEGG pathways such as sphingolipid signaling, chemokine signaling, Rap1 signaling, and cAMP signaling, besides others (Fig. 3a) were found to be significantly enriched among these 18 genes. Several of these pathways were also associated with S1PR3 and sepsis survival-related immune pathways (KEGG pathways) identified in Fig. 1. This strongly indicates that the S3MS signature builds a bridge between S1PR3 and sepsis survival-related genes. The protein and protein interaction (PPI) network analysis [22] provides biological insights such as the classification of protein complexes. Our results (Fig. 3b) from the PPI network analysis clearly suggests that the 18 genes can be grouped into two gene clusters. One gene cluster was associated with the VEGF signaling pathway, Rap1 signaling pathway, and Fc gamma R-mediated phagocytosis, while the other was associated with Staphylococcus aureus infection, chemokine signaling pathway, and cytokine–cytokine receptor interaction (Fig. 3b). Additionally, the correlation matrix demonstrates that 16 genes have a mostly positive correlation with each other while only 2 genes (CXCL10 and RAC1) demonstrated a negative correlation (Fig. 3C). Our results confirm that the 18 genes identified in our S3MS had a strong connection and relationship.

### S3MS predicts clinical outcomes in both discovery and validation cohorts

**Fig. 4:**
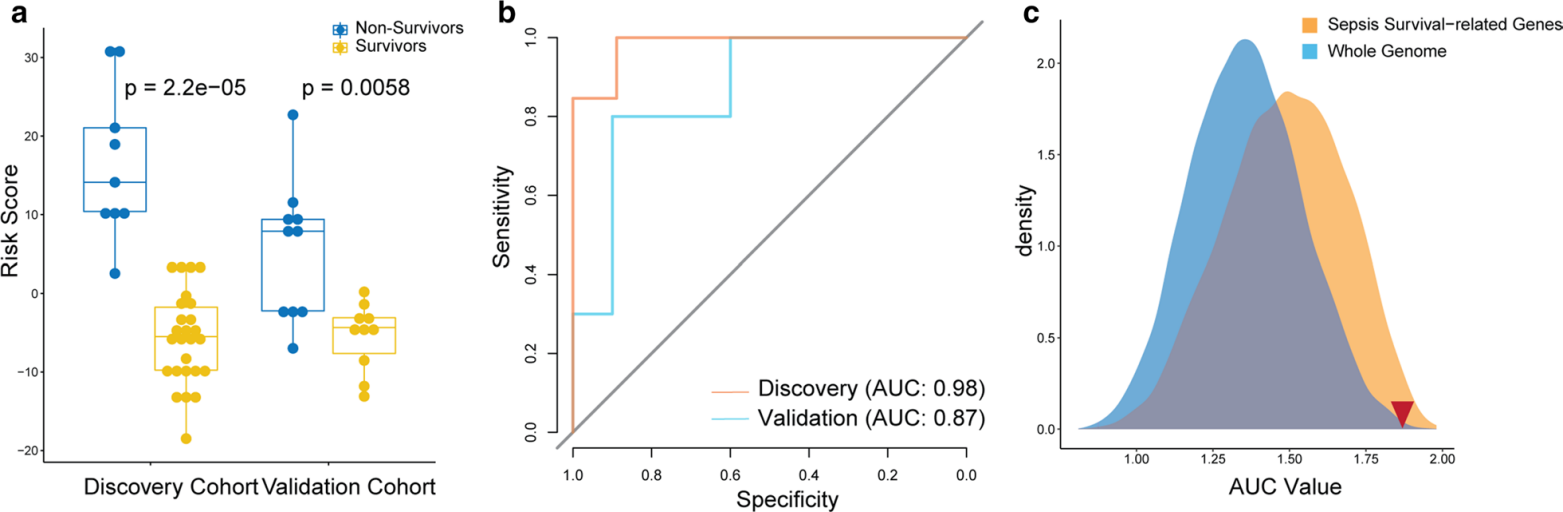
The S3MS-based sepsis risk score discriminates non-survivors from survivors. **a** Box plot of the S3MS-based risk scores in non-survivors and survivors. **b** ROC curves of the S3MS-based risk scores in distinguishing non-survivors from survivors. **c** Predictive power of the S3MS-based AUC values in the discovery and validation cohort compared with random gene signatures from the whole genome and sepsis survival-related genes

We developed a risk assessment scoring method to measure the possibility of sepsis risk in patients using a linear combination of the 18-gene expression values (Table 1). Each value was given a weighted value which indicates the direction of differential expression in non-survivors, and patients were assigned a score based on those measures. Our results focused on both the discovery and validated cohorts. The results are in line with our expectations: risk scores from non-survivors were significantly higher than those of survivors in our study (Fig. 4a). Therefore, our gene signature from S1PR3 has the potential to predict clinical outcomes in sepsis.

### Classification power of the 18-gene signature

We investigated the classification performance of the S1PR3 gene signature in the discovery and validated datasets. The AUC under the ROC curve were 0.998 and 0.8 for the discovery and validation cohorts, respectively (Fig. 4b).

A bioinformatics study by Venet et al. shows that most gene signatures randomly selected from the human genome with the same gene size were sometimes better than published gene signatures [23]. We collected 10,000 random gene signatures through random selection from the whole genome or sepsis survival-related genes and produced either an expression score for the entire genome or a risk score for the sepsis survival-related genes for each patient. Corresponding AUC values were then calculated for each random gene signature. Our gene signature had better power on the classification of sepsis survival than randomly generated genes with the same gene count (better than 95% percent random gene signatures in the whole genome) (Fig. 4c).

**Fig. 5:**
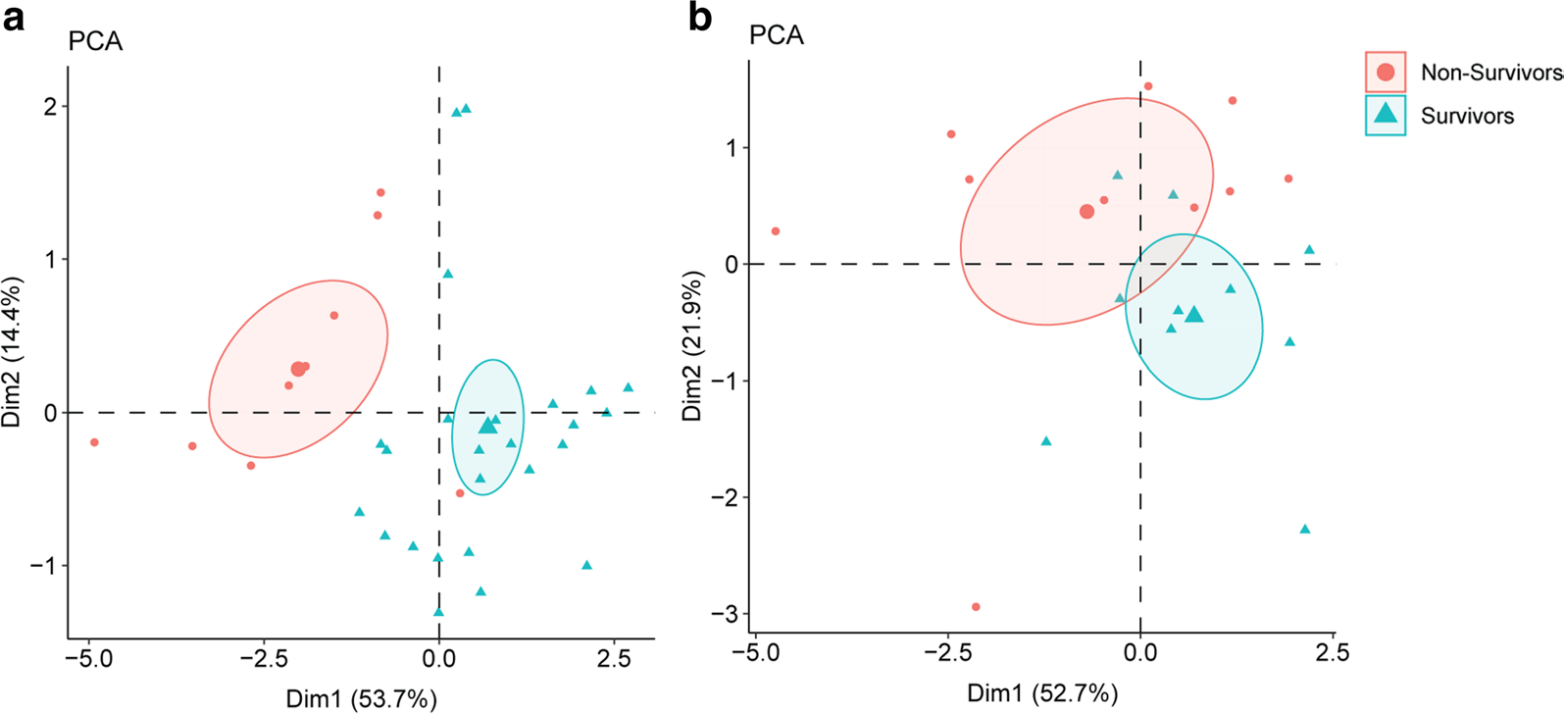
PCA plot of S3MS for the discovery cohort (**a**) and validation cohort (**b**)

Principal component analysis (PCA) was also performed to simplify the complexity in high-dimensional data like our 18-gene expression pattern. PCA (Fig. 5a, b) showed that the 18-gene signature thoroughly distinguished non-survival patients from survival patients in the discovery cohort, and only slightly overlapped in the validation cohort.

Overall, our 18-gene had a great classification power of discriminating the non-survivors from survivors on a gene expression level.

## Discussion

Sepsis is a systemic inflammatory response to pathogenic microorganisms such as gram-positive or gram-negative bacteria [24]. The mortality rate of sepsis is still relatively high (20% and 80% [25, 26]) despite improvement in sepsis treatment. Regulation of the immune response plays a vital role in the pathogenesis of sepsis [27, 28]. S1PR3, our target molecule, belongs to a family of GPCRs that regulate host innate immunity. Jinchao Hou et al. [18] validated that S1PR3 expression in macrophages is up-regulated following bacterial stimulation and ameliorated the severity of sepsis. Additionally, S1PR3 regulates various inflammatory responses and vascular barrier function in several pathological septic conditions [29]. S1PR3 is an attractive biomarker candidate due to its involvement in several signaling pathways and gene co-expression related to sepsis. Finding S1PR3 gene signatures in sepsis will reveal several biological or clinical features of the pathogenesis and progression of sepsis. In this study, we chose two gene expression datasets from the GEO database which contain whole blood samples from sepsis patients with clinical outcome information to identify an S1PR3 gene signature. Our investigations yielded the following observations: (1) Identification of an 18-gene signature in this study which could discriminate non-survivors from survivors at the gene expression level. (2) 18-gene signature is an “independent” prognostic marker for predicting or estimating the potential risk of sepsis. (3) Signaling pathways enriched in our gene signature linked S1PR3 pathways with severe sepsis-related processes.

Biomarkers are a non-invasive clinical method that could objectively predict or evaluate usual biological processes or host response to therapy. Up to now, the use of molecular biomarkers has been only concentrated on the diagnosis of sepsis, no gene signatures are used in the prognosis of severe sepsis. No biomarkers with single genes are likely to adequately reveal the rapidly evolving nature of a septic patient’s status. Some studies [6, 7] have attempted to combine several pro-inflammatory biomarkers or both pro- and anti-inflammatory markers randomly. However, we specifically derived the 18-gene signature based on the S1PR3 processes which are strongly related to the development of sepsis. Our gene signature performs very well as a novel biomarker for sepsis survival based on the performance of our risk assessment score in both the discovery and validation cohorts. This novel biomarker exhibits significance in three ways: (1) Independence. Our S3MS was not derived from a combination of other biomarkers, so it is a method to discover new sepsis biomarkers based on key signaling pathways; (2) Performance. S3MS performs not only better than random gene signatures from the whole genome with the same size but better than sepsis survival gene signatures with the same gene size (see Fig. 4). (3) Growth potential. Unlike most biomarkers such as circulating protein, gene signature can be adjusted dynamically to functionally perform better.

In order to account for the potential for false-positive results that are typical of multiple-testing [23], we have utilized random gene signature comparison. Therefore, resampling tests were utilized in our study in discovery and validation cohorts to answer this doubt. S3MS-based AUC values in the discovery and validation cohort were compared with random gene signatures from the whole genome and sepsis survival-related genes. The results showed that the classification power of the 18-gene signature is mostly better than that of the gene sets randomly chosen from the whole genome. Comparing to random genes or combined biomarkers, our S3MS also demonstrated a strong relationship with signaling pathways and protein interactions.

Numerous signaling pathways have been identified as enriched within our gene signature. The VEGF (vascular endothelial growth factor) pathway, which regulates vascular development in angiogenesis, was enriched in our gene signature. Kiichiro et al. [30] highlighted the importance of the VEGF pathway in altering sepsis morbidity and mortality. Many innate or adaptive immune pathways (chemokine signaling pathway, cytokine–cytokine receptor interaction, and Rap1 signaling pathway) were enriched within our 18-gene signature. Down-regulation of these immune pathways is known to worsen the prognosis of sepsis. Otherwise, 18 genes had intense interactions with each other. Hence, the 18-gene signature from S1PR3-related genes not only predicted the clinical outcome of sepsis patients but also revealed the signaling pathways which could play a pivotal role in the development and progression of sepsis.

Among S1PRs 1–5, we found both S1PR1 [31] and S1PR3-related gene signature are capable of predicting the survival of patients with sepsis. Several studies [18, 32] already indicated that S1P-S1PR3 signaling drove bacterial killing, S1PR3 was associated with preferable sepsis outcomes. In contrast to S1PR3, S1PR1 didn't have corresponding experimental support, so the S1PR3-related signature holds more prognostic value than S1PR1. S1PR1 and S1PR3 play a diverse role and belongs to different signaling pathways in sepsis progression. S1P–S1PR1 signaling plays a critical role in supporting the integrity of the endothelial barrier, while S1P-S1PR3 signaling drives bacterial killing in macrophages. Only 4 common genes among S1PR1-associated genes (557 genes) and S1PR3-related genes (226 genes). In general, S1PR1 and S1PR3 gene signature have diverse molecular mechanisms in the pathology of sepsis, so we decided to publish them separately.

In this study, we showed that the S1PR3-related protein-coding gene signature is capable of predicting which patients are at an elevated risk of developing severe sepsis. However, our work in the S1PR3-related gene signature was only based on bioinformatics methods. The potential power of our gene signature needs to be verified by clinical investigations.

## Conclusions

In conclusion, we identified a gene signature containing 18 protein-coding genes capable of being reproducible predictors of clinical outcomes in patients with sepsis. Thus, our results could have a potential value in clinical evaluations and disease monitoring in patients with sepsis and may ultimately help improve sepsis treatment algorithms based on severity risk.

## Supplementary Information

**Additional file 1: **The list of S1PR3-related genes.

**Additional file 2:** Co-expression correlation r values of S1PR3 with S1PR3-related genes.

**Additional file 3:** The list of sepsis survival-related genes.

## Data Availability

The gene expression and clinical data of GSE54514 and GSE33118 were downloaded from GEO database, while the gene functional interaction data was downloaded from STRING database version 11.

## Abbreviations

AUC: Area under the curve
DEGs: Differentially expressed genes
GPCRs: G protein-coupled receptor
GEO: Gene expression omnibus
KEGG: Kyoto Encyclopedia of Genes and Genomes
PPI: Protein–protein interaction
PCA: Principal component analysis
ROC: Receiver operating characteristic
S1P: Sphingosine-1-phosphate
S1PR3: S1P receptor 3
S3MS: S1PR3-related molecular signature
